# The Primacy of High B-Value 3T-DWI Radiomics in the Prediction of Clinically Significant Prostate Cancer

**DOI:** 10.3390/diagnostics11050739

**Published:** 2021-04-21

**Authors:** Alessandro Bevilacqua, Margherita Mottola, Fabio Ferroni, Alice Rossi, Giampaolo Gavelli, Domenico Barone

**Affiliations:** 1 Department of Computer Science and Engineering (DISI), University of Bologna, Viale Risorgimento 2, I-40136 Bologna, Italy; 2 Advanced Research Center on Electronic Systems (ARCES), University of Bologna, Via Toffano 2/2, I-40125 Bologna, Italy; margherita.mottola@unibo.it; 3 Department of Electrical, Electronic, and Information Engineering “Guglielmo Marconi” (DEI), University of Bologna, Viale Risorgimento 2, I-40136 Bologna, Italy; 4 IRCCS Istituto Romagnolo per lo Studio dei Tumori (IRST) “Dino Amadori”, Via Piero Maroncelli 40, I-47014 Meldola, Italy; fabio.ferroni@irst.emr.it (F.F.); alice.rossi@irst.emr.it (A.R.); giampaolo.gavelli@irst.emr.it (G.G.); domenico.barone@irst.emr.it (D.B.)

**Keywords:** prostate cancer, radiomics, machine learning, tumor staging, cancer heterogeneity, image processing

## Abstract

Predicting clinically significant prostate cancer (csPCa) is crucial in PCa management. 3T-magnetic resonance (MR) systems may have a novel role in quantitative imaging and early csPCa prediction, accordingly. In this study, we develop a radiomic model for predicting csPCa based solely on native b2000 diffusion weighted imaging (DWI_b2000_) and debate the effectiveness of apparent diffusion coefficient (ADC) in the same task. In total, 105 patients were retrospectively enrolled between January–November 2020, with confirmed csPCa or ncsPCa based on biopsy. DWI_b2000_ and ADC images acquired with a 3T-MRI were analyzed by computing 84 local first-order radiomic features (RFs). Two predictive models were built based on DWI_b2000_ and ADC, separately. Relevant RFs were selected through LASSO, a support vector machine (SVM) classifier was trained using repeated 3-fold cross validation (CV) and validated on a holdout set. The SVM models rely on a single couple of uncorrelated RFs (ρ < 0.15) selected through Wilcoxon rank-sum test (*p* ≤ 0.05) with Holm–Bonferroni correction. On the holdout set, while the ADC model yielded AUC = 0.76 (95% CI, 0.63–0.96), the DWI_b2000_ model reached AUC = 0.84 (95% CI, 0.63–0.90), with specificity = 75%, sensitivity = 90%, and informedness = 0.65. This study establishes the primary role of 3T-DWI_b2000_ in PCa quantitative analyses, whilst ADC can remain the leading sequence for detection.

## 1. Introduction

Prostate cancer (PCa) is the most common malignancy diagnosed in men worldwide [1]. This strongly impacts clinical management in terms of costs and resources, also based on the PCa stage at the diagnosis that could suggest different clinical pathways [2]. Locating and discriminating clinically significant (csPCa) from non-significant cancer (ncsPCa) remain a challenge in PCa management. The definition of csPCa is a dynamic process initiated many years ago, when there was the first evidence of a great population of patients with a PCa diagnosed at autopsy without any clinical manifestations [3]. At present, csPCa is defined as the presence of any of the following: Gleason score (GS) ≥3 + 4, volume > 0.5 mL, extraprostatic extension. ncsPCa is defined as a cancer GS of 3 + 3 = 6 involving fewer than two cores at biopsy and <50% of any given core and prostate-specific antigen (PSA) density of <0.15 ng/mL per cm^3^; it generally has a favorable prognosis, with a high life expectancy at 10 years from diagnosis, and a low risk of biochemical recurrence [4]. Even if there is no consensus regarding the optimum management of localized disease, ncsPCa was adopted as components of the “very low-risk category” of the National Comprehensive Cancer Network guidelines in which active surveillance (AS) protocol is supported as a management option. AS is a strategy of close monitoring, typically using PSA, repeat biopsies and multiparametric magnetic resonance imaging (mpMRI), keeping curative treatment for those with evidence of disease. It has been recommended for men with low-risk disease. Instead, csPCa may be subjected to curative options that include prostatectomy (RP), external beam radiotherapy (RT) or low-dose-rate brachytherapy [5]. 

PCa aggressiveness is conventionally assessed through biopsy, that can be random or aimed at the most supposedly malignant areas, whether it is transrectal ultrasound (TRUS)- or MRI-guided [6]. Frequently, biopsy outcomes are reported to differ from those obtained after RP [7], and even between closely repeated examinations [8]. Moreover, notable side effects are experienced by men undergoing biopsy, including bleeding, pain and infection [9]. Therefore, the availability of non-invasive imaging approaches for distinguishing ncsPCa from csPCa is a very attractive prospect to increase the detection rate of csPCa and spare patients from unnecessary biopsies and overtreatment. 

mpMRI is employed in the clinical routine, primarily for PCa detection because it facilitates localization of PCa and can help in targeting prostate biopsy. The current guidelines of Pi-RADS v2.1 underline the key role of morphological T2-weighted sequence (T2w) and diffusion weighted imaging (DWI) to obtain functional information regarding variations of tissue diffusivity.

DWI sequences are sensitive to microscopic water motion in biological tissue and help differentiate normal from tumor tissue, where the structural change of the biological components and the hypercellularization processes hamper the motion of the water molecules [10]. Water restriction yields a high DWI signal, detecting tumor changes towards malignancy, progressively more emphasized in high b-value sequences (b ≥ 1000 s/mm^2^), to the detriment of the benign glandular components, where any morphological reference is lost. However, there are some limitations to qualitative assessment on DWI. In particular, the signal intensity (SI) depends on both water mobility and T2-relaxation time of tissue, so a lesion with very long T2 may demonstrate high SI on the DWI (T2-shining thought artefact); therefore, SI of different solid tumor may be similar [11]. In the clinical practice, a definite confirmation of real hyperintense signal DWI areas is conveyed by the apparent diffusion coefficient (ADC) maps. ADC maps are a reconstruction derived from a normalization process of two or more DWI sequences acquired at different b-values. High signals in DWI are converted into low signals in ADC maps, which recover the information related to the apparent diffusion of water’s molecules, thus losing the specific measures contained in the native DWI sequences, which arise directly from the tissue properties. Nevertheless, the ADC normalization process also allows removing the misleading high signals in DWI and, consequently, distinguishing the tumor boundaries more clearly, keeping the morphological information of the gland [10], albeit motion artefacts can remain and could alter the ADC map processing. Moreover, ADC maps implicitly have a higher signal to noise ratio (SNR) than the individual parent b-value images and, as such, they are currently one of the most effective sequences for PCa detection and localization [12]. Accordingly, ADC has been predominantly exploited in quantitative imaging as well ([13,14]), eventually combined with T2-weighted and native DWI sequences [9,15,16,17], and on several occasions, ADC metrics have proved to correlate with the GS successfully. It is not surprising that good results have been achieved by previous studies in classifying ncsPCa and csPCa [13,17].

At present, 3T-MRI systems enable the acquisition of high b-value DWI sequences with higher SNR, more reduced noise and more limited artefacts than lower-field MRI scanners. Consequently, with these systems, the motivations promoting the main use of ADC, relegating the DWI to a secondary role, could decay. The present technology allows exploiting the native 3T-DWI sequences at their best, both in the clinical practice and, above all, in the quantitative imaging. 

It is widely debated in the literature what the best b-value for prostate cancer detection could be in order to highlight the tumor tissue, reducing the signal from the surrounding benign tissue. However, b = 2000 s/mm^2^ of a 3T system is expected to be the most appropriate [18,19] because it can embody quantitative information regarding tissue heterogeneity and tumor functional properties with specificity and sensibility higher than ADC.

In our study, we investigate the effectiveness of DWI_b2000_ sequences in quantitative tissue characterization through a predictive radiomic model developed to detect csPCa in patients with GS > 3 + 3, exploiting only image-based features, also compared with ADC performing the same task.

## 2. Materials and Methods

### 2.1. Patient Cohort

This retrospective study enrolled patients between January–November 2020 with a clinical confirmation of PCa undergoing mpMRI, all having DWI acquisition protocol including DWI_b2000_. All patients eligible for this study underwent TRUS biopsy performed as part of standard-of-care [20] or due to recruitment into clinical trials at our institution. Eighteen-core biopsy was performed six weeks before mpMRI. In a few cases, mpMRI was performed before the term of six weeks due to urgent clinical need regarding preoperative patients. In these cases, if a prominent hemorrhage was detected, patients were not included in the study. In addition, patients with hip prosthesis were not included in the study. Thus, 105 patients were enrolled, among which fifteen were excluded because of previous administration of RT or focal therapies, eight underwent asynchronous execution of TURP and six presented severe motion artefacts. Finally, 76 patients were included. This retrospective study received IRB approval and written informed consent was waived. Based on biopsy outcome, fifty patients with GS ≥ 3 + 4 were referred to as csPCa and twenty-six patients with GS = 3 + 3 were considered ncsPCa. Table 1 reports detailed clinical parameters of patients included in this study, such as PI-RADS score, location of PCa lesions and PSA level surveyed contextually to mpMRI.

### 2.2. mpMRI Protocols

Images were acquired with a 3T multicoil Ingenia MRI system (Philips). mpMRI protocols include T2-weighted (T2w), DWI, ADC maps and dynamic contrast enhanced MRI (DCE-MRI) sequences. In this regard, it is worth mentioning that, for scientific aims, all DWI sequences were previously acquired employing nine different b-values and ADC maps referred to all of them, accordingly. Patient preparation required fasting 6 h before the examination, bowel preparation to be performed 2 h before the examination and emptying of the bladder. To reduce peristaltic motion, 1 mL of scopolamine–butylbromide (Buscopan, Boehringer Ingelheim, Ingelheim, Germany) was administered in a slow bolus infusion at 20 mg/mL, diluted in 10 mL of saline solution. Table 2 reports details of DWI protocols for the seventy-six patients included in this study.

### 2.3. PCa Lesion Segmentation

**Figure 1:**
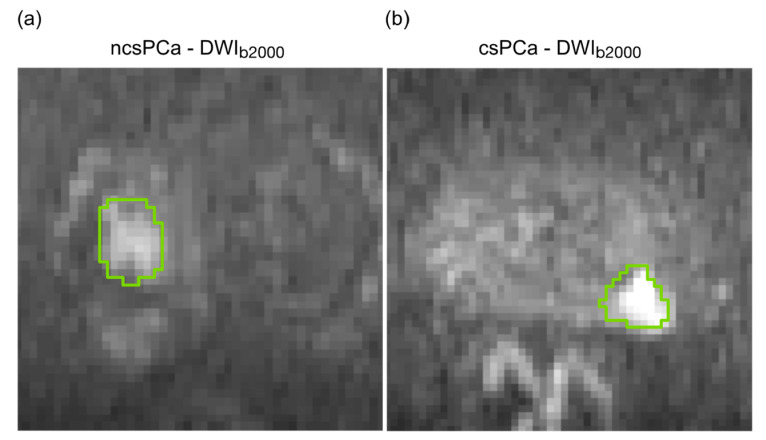
(**a**) ROIs of PCa lesions outlined on DWI_b2000_ for a representative ncsPCa; (**b**) ROIs of PCa lesions outlined on DWI_b2000_ for a representative ncsPCa.

MRI examinations were analyzed in consensus by two radiologists with twenty-five (**) and seven-year (**) experience in urogenital pathologies. Axial T2w, DWI, DCE sequences and ADC maps were considered contemporarily for reporting and each detected lesion was assigned a PI-RADS score [4]. Using cognitive fusion of all available MRI sequences, PCa lesions were manually segmented on DWI_b2000_ using Aliza Medical Imaging 1.98.18 (Bonn, Germany—https://www.aliza-dicom-viewer.com/ (accessed on 11 September 2020) [21]). All PCa lesions having at least a PI-RADS 3 were outlined slice by slice along the most emphasized internal boundaries. While PCa lesions in the peripheral zone (PZ) were segmented directly on DWI sequences, for central and transitional zone, lesion ROIs were outlined on DWI_b2000_ and refined using the cognitive fusion of parallel axial T2w images. Figure 1 shows the lesion ROIs outlined on DWI_b2000_ for two representative ncsPCa (Figure 1a) and csPCa (Figure 1b). 

Then, the regions of interest (ROIs) were reported on ADC maps due to the natural coregistration of ADC with its parent DW images.

### 2.4. Radiomic Feature Extraction

RFs were extracted from PCa ROIs, from both ADC and DWI_b2000_ sequences. For each slice with lesion, seven first-order RFs, including mean, median, skewness, kurtosis, interquartile range, coefficient of variation [22] and entropy, were computed on a local tissue patch based on the method proposed in [22,23], in order to account for the small changes of tissue heterogeneity occurring between neighbor voxels. The smallest informative tissue unit for radiomic analysis was chosen to be approximately 1 cm^2^. Hence, the size of the local patch has been set stemming from the different resolutions of the examinations (Table 2), to explore a minimum distance from the central pixel of 0.5 cm along the vertical and horizontal directions, here corresponding to a square window with side varying from five to seven pixels. In practice, for each ROI’s pixel, seven distribution of first-order RFs were first computed, considering the surrounding pixels of a square patch centered on the pixel itself. Then, on each of these seven distributions, twelve global RFs were computed (i.e., maximum value, standard deviation, median absolute deviation, mean and median values of the last decile, besides the seven abovementioned RFs), thus finally yielding 84 RFs. The mathematical formulation of all RFs is provided in Electronic Supplementary Material 1 (S1). RFs’ extraction together with the subsequent predictive model building and data analysis were performed in MATLAB^®®^ (R2019b v.9.7, The MathWorks, Natick, MA, USA).

### 2.5. Predictive Model 

**Figure 2:**
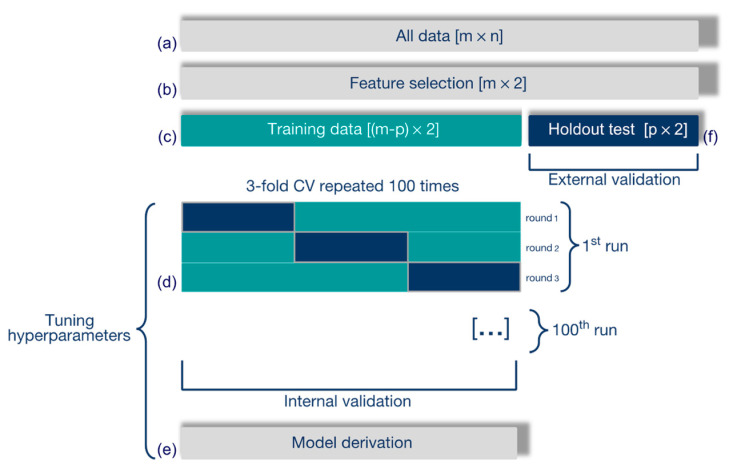
Development of the radiomic model to predict csPCa. (**a**) RFs are normalized and standardized, and (**b**) selected through LASSO. (**c**) A linear SVM classifier is trained and (**d**) 3-fold CV is performed for internal validation. (**e**) The final model is selected and (**f**) externally validated on the holdout test set.

A radiomic model was built to recognize csPCa (true positives, TPs), distinguishing them from ncsPCa (true negatives, TNs), according to the process outlined in Figure 2. 

All RFs (Figure 2a) were normalized and standardized, and redundant and irrelevant RFs were removed through the least absolute shrinkage and selection operator (LASSO), with the optimal tuning parameter (λ) selected using 10-fold cross validation (CV, Figure 2b) and the minimum CV error rule. To prevent overfitting, only two RFs were considered from the subset of RFs selected from LASSO. First, the couples with a high Pearson correlation (ρ ≥ 0.15) were discarded. Second, the most discriminant couple of RFs (i.e., yielding the lowest *p*-value according to the Wilcoxon rank-sum test, corrected with Holm–Bonferroni) was selected from those surviving the previous step. 

The entire data set was split into training and (holdout) test set, made up of 48 and 28 patients, respectively. The training set consisted of 18 ncsPCa and 30 csPCa, whilst the test set comprised 8 ncsPCa and 20 csPCa. To preserve the representativeness of the training set without degrading the generalization performance, the training set has been derived from the entire dataset to include the patients’ candidate for representing the support vectors (SVs) of an SVM classifier, according to the method described in [24], based on their distance from the separating hyperplane. Then, the SVM classifier with linear kernel was trained on the training set (Figure 2c) with a 100-time repeated 3-fold CV, (Figure 2d) for tuning the SVM hyperparameters, that is, the kernel scale (ɣ) and the global misclassification cost (C). C was then scaled by the weight of the error occurring in each class, which corresponded to its own prior probability [25]. Then, a binomial logit function was used to compute, from each SVM trained model, the predicted class for each patient and the corresponding probability score, this representing the final radiomic score. Each CV-fold was made up of sixteen patients, six ncsPCa and ten csPCa. To prevent any spurious solution, an internal validation procedure was performed by one hundred repetitions of 3-fold CV. For each round, the receiving operating characteristic (ROC) curve and the corresponding area under the curve (AUC) were computed for training and validation sets. Then, for each run, the SVM models most prone to overfitting, yielding an AUC on the validation set higher than that on the training one, were discarded, while the highest F2-score computed on the validation sets of remaining models, if any, selected the best one [26]. Finally, at most 100 SVM models survived and an early selection was carried out by analyzing their performance on the training sets, discarding the models with a very low C parameter (C < 1), more prone to overfitting and with F2-score < 0.80. At the end, the model showing the highest F2-score on the validation set (Figure 2e) was selected as the ultimate predictive model, to be externally validated on the holdout test set (Figure 2f). The performance of the SVM classifier was assessed through AUC, and sensitivity, specificity and informedness (I) were measured at the Youden cutoff. The positive predictive values (PPV) and false detection rate (FDR) were computed accordingly.

The same procedures were carried out for building both the predictive models (based on either ADC or DWI_b2000_ sequences). 

## 3. Results

### 3.1. ADC Model

**Figure 3:**
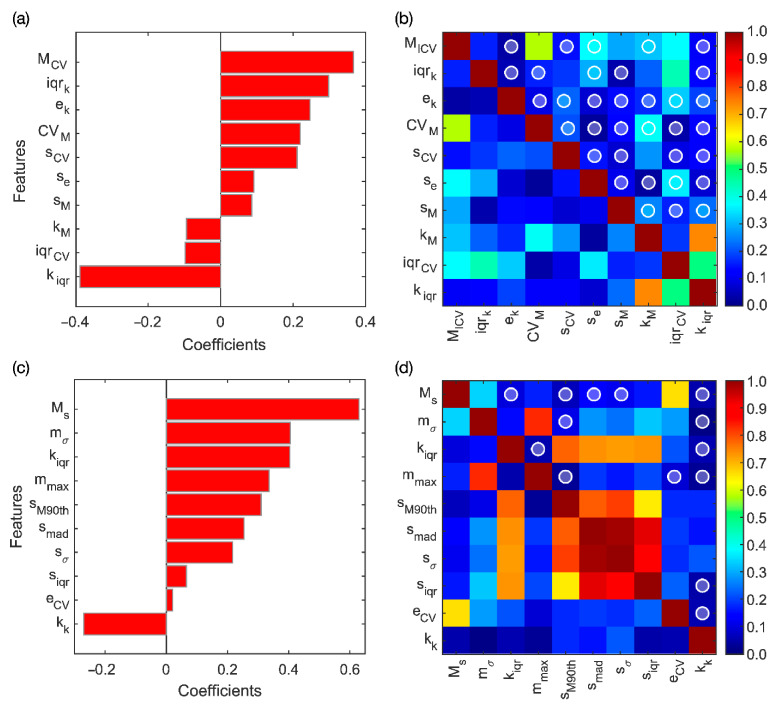
(**a**) Coefficients of the ten RFs selected through LASSO for ADC and (**b**) their correlation matrix. (**c**) Coefficients of the ten RFs selected through LASSO for DWI_b2000_ and (**d**) their correlation matrix. In (**b**,**d**), the white circles highlight the uncorrelated couples (ρ < 0.15).

LASSO yielded ten relevant RFs, which are reported in Figure 3a according to their rank. 

The correlation coefficients computed between all the ADC-based RF couples are resumed in the matrix shown in Figure 3b, where the white-outlined circles highlight thirty-four uncorrelated couples arising from the LASSO selection. Six significant RF couples resulted significant in Wilcoxon rank-sum test, with *p*-value ≤ 1.4∙10^−3^ after considering Holm–Bonferroni correction. The most discriminant RF couple (*p*-value~10^−4^) is composed by the coefficient of variation of the median (M_CV_) and the interquartile range of the kurtosis (k_iqr_), whose LASSO coefficients are 0.367 and −0.388, respectively, corresponding to the most powerful positive and negative RFs, respectively. Basically, the selected RFs provide different measures of local variability of diffusivity restriction. 

**Figure 4:**
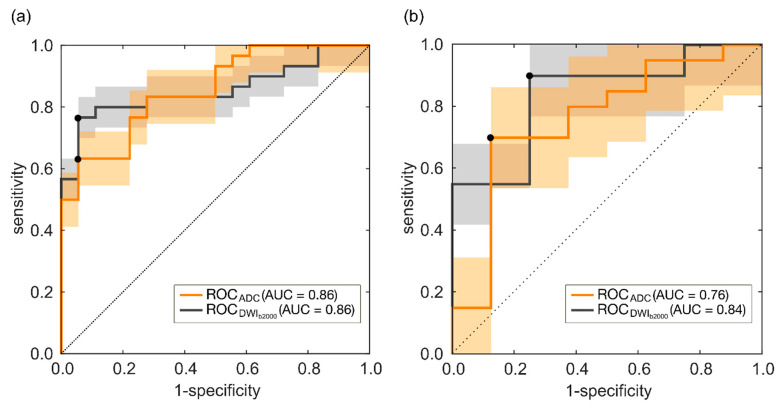
(**a**) ROC curve achieved on the training set for ADC and DWI_b2000_ models. (**b**) ROC curves achieved on the holdout test set for ADC and DWI_b2000_ models.

In the training set, the couple M_CV_-k_iqr_ predicts csPCa according to the ROC reported in Figure 4a, with AUC = 0.86 (95% CI, 0.74–0.91), and sensitivity and specificity at the Youden cutoff (I = 0.58) equal to 63% and 94%, respectively. 

Hence, prediction of csPCa is achieved in the training set with 11 FN and 1-only FP, thus yielding FDR = 0.05, PPV = 0.95, with F_2_-score = 68%. Figure 4b shows the ROC of the couple of RFs M_CV_-k_iqr_ achieved for the holdout test set, with AUC = 0.76 (95% CI, 0.63, 0.96) and sensitivity and specificity at the Youden cutoff (I = 0.58) equal to 70% and 88%, respectively. Hence, referring to the holdout test set, the prediction of csPCa is achieved with 6 FN and 1-only FP, with FDR = 0.07, PPV = 0.93 and F_2_-score = 0.74.

### 3.2. DWI_b2000_ Model

LASSO yields ten relevant RFs, whose coefficients are reported in Figure 3c according to their rank. The correlation coefficients computed between all the RF couples are resumed in the matrix shown in Figure 3d, where the white-outlined circles highlight fourteen uncorrelated couples. Eleven of them resulted in significance at Wilcoxon rank-sum test, with *p*-value ≤ 0.0125, after considering Holm–Bonferroni correction. The most discriminant RF (*p*-value~10^−7^) is composed by the standard deviation of the mean, m_σ_, and the median of the last decile of the skewness, s_M90th_, whose LASSO coefficients are 0.405 and 0.310, respectively, corresponding to the second and the fifth RFs. The selected RFs give information regarding the heterogeneity and the degree of asymmetry of local cellularity values measured at DWI_b2000_.

**Figure 5:**
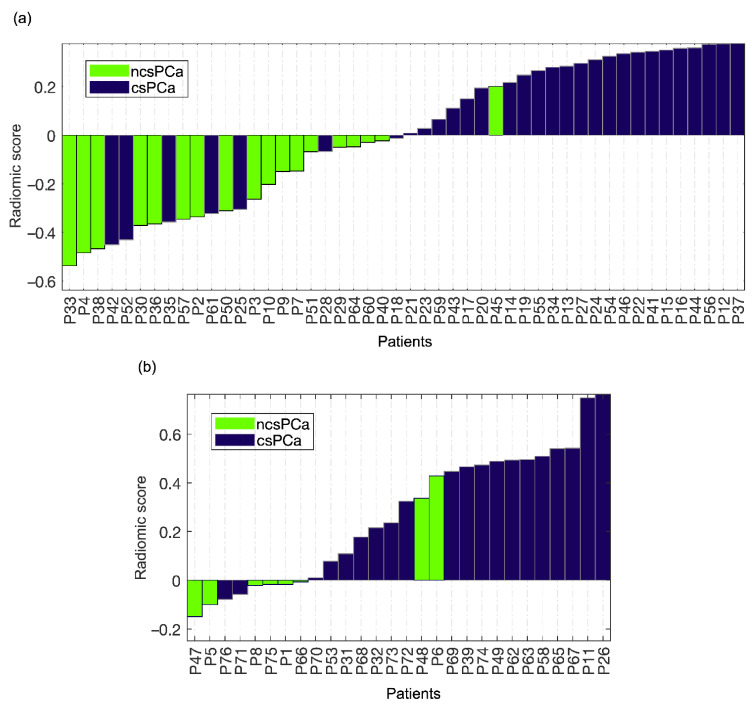
(**a**) Waterfall plot achieved for the predictive model based on DWI_b2000_ on the training set. (**b**) Waterfall plot achieved for the predictive model based on DWI_b2000_ on the holdout test set.

In the training set, the couple m_σ_-s_M90th_ can predict csPCa according to the ROC reported in Figure 4a, with AUC = 0.86 (95% CI, 0.79–0.93) and sensitivity and specificity at the Youden cutoff (I = 0.71) equal to 77% and 94%, respectively. Figure 5a also reports the waterfall plot of the radiomic score computed for each patient based on the couple m_σ_-s_M90th_, where ncsPCa and csPCa are highlighted with green and dark blue bars, respectively. 

**Figure 6:**
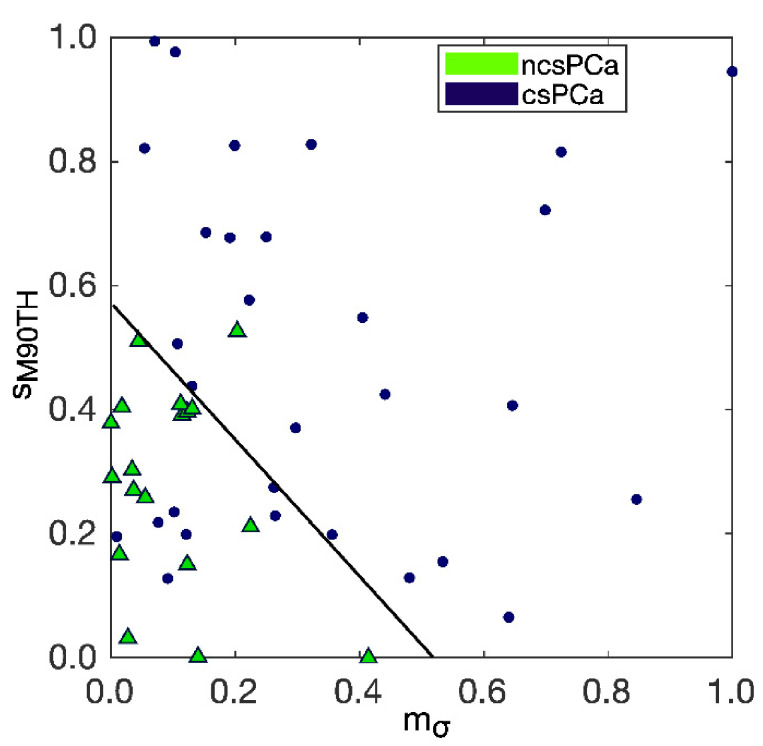
Separation between csPCa and ncsPCa performed by the trained SVM classifier referring to DWI_b2000_, with the separation hyperplane highlighted in black.

Hence, in the training set there are 7 FN and 1-only FP, with FDR = 0.04, PPV = 0.96 and F2-score = 0.80. The separation between csPCa and ncsPCa performed by the trained SVM classifier is also shown through the scatter plot in Figure 6, where the separation hyperplane is highlighted in black. 

**Figure 7:**
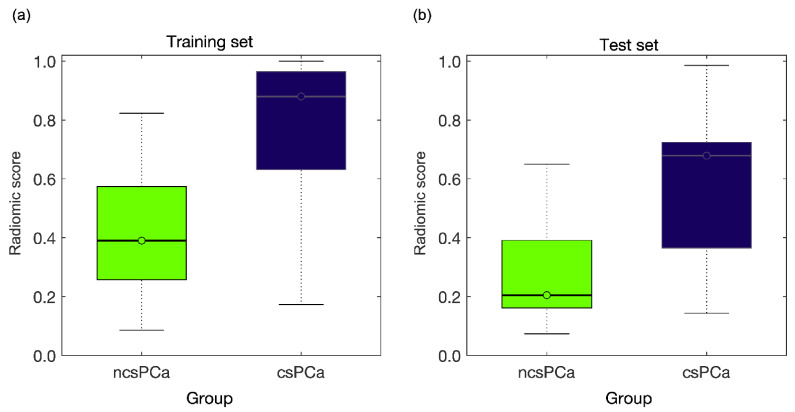
(**a**) The boxplot of the separation between ncsPCa (light green box) and csPCa (dark blue box) for the training set where the two groups are separated with a *p*-value~10^−5^. (**b**) The boxplot of the separation between ncsPCa (light green box) and csPCa (dark blue box) for the test set, where the two groups are separated with *p*-value = 7∙10^−3^.

Figure 4b shows the ROC of the couple of RFs m_σ_-s_M90th_ achieved for the holdout test set, with AUC = 0.84 (95% CI, 0.63, 0.90) and sensitivity and specificity at the Youden cutoff (I = 0.65) equal to 90% and 75%, respectively. Figure 5b shows the waterfall plot referring to the holdout test set, where prediction of csPCa is achieved with 2 FP and 2 FN, FDR = 0.10, PPV = 0.90 and F2-score = 0.90. The boxplot of the separation between ncsPCa (light green box) and csPCa (dark blue box) is shown in Figure 7 for the training (Figure 7a) and the test sets (Figure 7b), respectively. 

In the training set the two groups are separated with a *p*-value~10^−5^, this reflecting the great difference between the median values of the radiomic score of the two groups, 0.39 for ncsPCa and 0.88 for csPCa. Similarly, in the holdout test set, the two groups are separated with *p*-value = 7∙10^−3^, and the median values of the radiomic scores of ncsPCa and csPCa are 0.20 and 0.68, respectively.

## 4. Discussion

Biopsy examination is presently the reference clinical tool for distinguishing csPCa from ncsPCa, which allows for starting different clinical paths, that is, curative treatments or active surveillance, watchful waiting and observation, respectively [27]. mpMRI has an increasingly crucial role in prebiopsy patient management, to prevent patients undergoing unnecessary operations [15] which are known to cause side effects in about 30% of men, 1% of which requires hospitalization for observation [28]. A radiomic and quantitative mpMRI-based imaging approach is frequently adopted in PCa study with the aim of enriching the radiological assessment of medical images and providing additive information referring to tumor aggressiveness and prognosis, for instance, to distinguish csPCa from ncsPCa prior to biopsy. However, a “considerable overlap between csPCa and ncsPCa in mpMRI parameter values” is known [14] and it represents the major limitation for mpMRI to replace the biopsy in patient staging [14]. At present, ADC is still considered to be the most promising sequence for quantitative image analysis. In particular, the ADC images have been very successful in the clinical routine, mainly for two reasons. On the one hand, they allow reconstructing the diffusion-weighed information, achieving an SNR much higher than that of native DWI. On the other hand, they allow preserving the morphology, especially if compared to high b values, and annulling the artefacts of DWI images, such as the T2 shine artefact, which are known to mislead the assessments of suspicious malignant areas. Consequently, the ADC sequences have become the reference ones for confirming diagnosis of PCa and, as such, they have even been largely employed to extract information as regards PCa prognosis. To this purpose, let us consider the scientific works from PubMed database, published since 2015 and reported in Table 3, which implement a predictive model of csPCa (independently of the lesion zone). It is clear that all these works except [29] utilize the ADC sequence [13], sometimes coupled with T2w ([9,15,16,17]), whilst only one work combines ADC with IVIM parametric maps [14]. However, also in this last case, the best result reported refers to the mean value of the ADC map (ADC_mean_). 

As a matter of fact, high b-value DWI has already proved to increase both reader’s sensitivity [30] and radiomic accuracy in distinguishing PCa from non-cancerous lesions [31], albeit a limited success is reported in recognizing csPCa and ncsPCa so far. The authors in [14] even state that DWI sequences are not feasible yet for reliable clinical indications of tumor prognosis and, besides that, they cannot bring any added value with respect to the ADC sequence in identifying csPCa. On the contrary, the predictive model developed in this study on the basis of DWI_b2000_ only notably improves the prediction of csPCa, with PPV = 96% in the training set and PPV = 90% in the holdout test set, with respect to the clinical mpMRI used in triage prebiopsy setting reaching at most PPV = 51% [32]. At the same time, our radiomic model substantially bounds the risk of overtreatment, which results in it being only 4% in the internal validation sets and 10% in the external one, thus confirming the high potential role of radiomic MRI in clinical decision making. In fact, overtreatment of ncsPCa is reported as being the major side effect of the high-sensitivity tests used for revealing the tumor malignancy degree [33]. Moreover, boxplots in Figure 7 show that our results based on one RF couple extracted from DWI_b2000_ yield a wide separation between the two groups of ncsPCa and csPCa. The primacy of DWI_b2000_ in extracting quantitative information correlating with tumor aggressiveness is confirmed when analyzing the outcomes of the predictive model developed using the ADC. In fact, the performance of the ADC model is significantly lower than that of DWI_b2000_, albeit being in line with the results of the literature, detailed in Table 3. In practice, with the coming of the 3T MR systems there is no further need to limit the quantitative analysis of tissue diffusivity to ADC sequences only, and above all, quantitative information extracted by DWI_b2000_ is much more effective to characterize PCa than that derived by ADC. 

Comparing in detail the performance of our model with the works reported in Table 3, one can see that the work of [14], where the classification is performed exclusively with ADC_mean_, computed between b = 0 and b = 900 s/mm^2^, reports almost the worst values of AUC (AUC = 0.79) with I = 0.59. 

Analogously, [29], the only work using the DCE-MRI, reaches at most AUC = 0.75, the worst considered, with I = 0.56, substantially confirming the direction of the present guidelines PI-RADS v2.1, where “DCE-MRI has become secondary to DWI and T2w images”, also considering that prostate DWI has “ease of acquiring and processing the images in comparison with other functional MR techniques” [30]. In fact, two of the works considered, the first one employing ADC_mean_ [13] and the second one a radiomic signature where 7 out of 10 RFs are extracted by the ADC map [17], achieve quite high AUC values. In fact, AUC = 0.85 in [13] and AUC = 0.88 in [17], albeit with low I’s, I = 0.58 and I = 55, respectively, somewhat lower than ours (I = 0.65). Two works only include some native DWI sequences for extracting the radiomic signature, with b = 1500 in [9] and b = 0, 1000 in [16]. However, although the work in [9] reports a good AUC = 0.82 value, but I = 0.57, only one out of the nine features composing the signature is extracted from the DWI sequence, and it is not even the most important one. In addition, in [16], where the signature is made by ten RFs, and only five of them are extracted from DWI, a quite high AUC = 0.81 value is coupled with the worst I result (I = 0.53). Finally, [15] seems to achieve a result quite similar to ours in terms of AUC = 0.83, but no other metric is provided to perform a deep comparison. On the whole, it seems that ADC, although being largely employed, cannot offer the performance of DWI in detecting csPCa. This is due to the ADC parametric maps arising from a normalization procedure between DWI images at different b-values. In fact, normalization implicitly yields a low-pass (average) filtering of the local value differences between adjacent structures, thus weakening the native information conveyed by the original DWI sequences. In many works, DWI has been reported as “the best monoparametric component of prostate MRI assessment” [17], where “quantitative analysis at high b-value DWI” (from b = 1000 to b = 2000 s/mm^2^) “suggests” the highest sensitivity of DWI in both detecting PCa [30] and staging high-grade diseases [34], but it has had a limited diffusion in radiomic studies so far. We agree that visual-based tumor detection and segmentation can be performed with much higher accuracy on the ADC sequences, and these should remain the reference tool for visual assessments and ultimate confirmation of cancer diagnosis. Nonetheless, our results and some literature strongly suggest that they cannot be the best tool for quantitative imaging, since the information extracted is far beyond what even expert eyes can visually detect. Accordingly, the native DWI information can have a higher specificity, from a quantitative point of view, in detecting/catching the cellular differentiation degree needed to distinguish csPCa from ncsPCa. The authors of [17] report that the good performance of the radiomic model and of the ADC_mean_ are equivalent. Furthermore, based on our results, this suggests that a radiomic analysis carried out on DWI images rather than on ADC maps can yield a marked advantage, whether the original information is either visual or semi-quantitative. 

One final consideration is worth being reported. Often, the signal restriction in ADC has been attributed to the hypercellularity process associated, in its turn, with a progression in terms of tumor aggressiveness. In fact, the work of [10] shows how the ADC signal restriction is only weakly correlated to the main cell metrics (nuclear count, nuclear area), but the stronger correlation is reported with the variation of gland component volumes (epithelium, stroma and lumen). The tumor progression attributed to a higher GS results in being associated with an increasing volume of low-diffusivity epithelial cells and decreasing volumes of high-diffusivity stroma and lumen space. Accordingly, Gleason grade definitions rely on changes of tissue architecture, which make the tumor progressively more heterogeneous and less differentiated as malignancy increases. Thus, it is worth noting that our two RFs extracted from DWI_b2000_ are two direct measures of tissue asymmetry and local variability in tissue diffusivity. DWI_b2000_ seems to catch with high specificity the asymmetry gradients found between the local property of tissue diffusivity, following the disproportion between the gland components [10].

The main limitation of the study is inquiring into the role of DWI_b2000_ only in predicting csPCa, while other b values (e.g., b = 1200 or 1400 s/mm^2^) could also work, this being a matter for further investigations. Second, no clinical parameter (e.g., prostate volume, PSA, PSA density) has been addressed, since this requires a wider dataset, besides being beyond the scope of this research. Third, only PCa lesions with PI-RADS ≥ 3 have been included; in order to have mpMRI examinations showing PCa suspicions clear enough to train a predictive model. However, inclusion of PI-RADS 2 lesions would be useful in the first-line triage test in men with suspected cancer, worthy to be considered for a future study design.

## 5. Conclusions

In conclusion, our findings, to be confirmed in more extensive studies, assign the 3T-DWI_b2000_ sequence a primary role in quantitative analyses of PCa, useful for prognosis and targeting biopsy, while confirming the ADC as the leading sequence for detection. The ability to identify men with csPCa early remains a hot topic under active investigation. Accordingly, our study promoting a wider employment of 3T-DWI_b2000_ represents a marked step forward.

## Figures and Tables

**Table 1:** Clinical parameters of the study population, including age, PSA level surveyed contextually to mpMRI, location of PCa lesions, PI-RADS score and GS.

Study Parameters	ncsPCa	csPCa
No. of patients	26	50
Mean ± SD		
Age (years)	65 ± 8.8	66 ± 6.8
PSA (ng/mL)	5.30 ± 2.97	7.80 ± 7.48
Range		
Age (years)	[42÷78]	[48÷79]
PSA (ng/mL)	[0.80÷12.20]	[0.38÷37.00]
Lesions’ location		
PZ	25	64
TZ	-	1
CZ	8	10
PZ-TZ ^1^	2	1
PZ-CZ ^1^	1	3
AFS	-	1
No. of lesions per PiRADS score		
PI-RADS 3	16	15
PI-RADS 4	16	34
PI-RADS 5	4	33
No. of lesions per GS		
GS 3 + 3 (ISUP 1)	26	
GS 3 + 4 (ISUP 2)	-	22
GS 4 + 3 (ISUP 3)	-	14
GS 4 + 4 (ISUP 4)	-	8
GS 4 + 5 (ISUP 5)	-	4
GS 5 + 5 (ISUP 5)	-	2

^1^ Partial overlapping between zones.

**Table 2:** DWI acquisition protocol for the seventy-six patients included in the study.

DWI Protocol	
Coil	Multicoil
TR ^1^ (ms)	[3000, 5804]
TE ^1^ (ms)	[80, 87]
No. of slices ^1^	[24, 33]
Slice thickness (mm)	3
Slice gap (mm)	3
b values (s/mm^2^)	0, 50, 100, 150, 200, 250, 800, 1500, 2000
No. of gradients	3
Field of view ^1^ (mm^2^)	[160, 260]
Acquisition matrix ^1^	[96, 176]
Pixel spacing ^1^ (mm)	[1.41, 1.67]

^1^ Range.

**Table 3:** Comparison of our findings with the scientific works published since 2015 (from PubMed database) predicting csPCa (independently of the lesion zone).

Year	Author	mpMRI Sequences	Features	AUC	SE	SP	I
2015	Fehr et al. [15]	T2w, ADC	18 RFs	0.83	-	-	-
2017	Barbieri et al. [14]	ADC, IVIM	ADC_mean (*b*[0–900])_	0.79	0.85	0.74	0.59
2018	Bonekamp et al. [17]	T2w, ADC	10 RFs	0.88	0.97	0.58	0.55
2019	Cristel et al. [29]	DCE-MRI	K_trans_	0.75	0.95	0.61	0.56
2019	Min et al. [9]	T2w, ADC, DWI_b1500_	9 RFs	0.82	0.84	0.73	0.57
2020	Zhang et al. [16]	T2w, ADC, DWI	10 RFs	0.81	0.80	0.73	0.53
2020	Hiremath et al. [13]	ADC	ADC_mean (b[0–1300])_	0.85	0.77	0.81	0.58
**2021**	**Our study**	**ADC**	**2 RFs**	**0.76**	**0.70**	**0.88**	**0.58**
**2021**	**Our study**	**DWI_b2000_**	**2 RFs**	**0.84**	**0.90**	**0.75**	**0.65**

The comparison is based on mpMRI sequences adopted, number of RFs, AUC values, sensitivity (SE), specificity (SP) and informedness (I).

## Data Availability

The data are not available because of patients’ privacy.

## Supplementary Materials

The following are available online at https://www.mdpi.com/article/10.3390/diagnostics11050739/s1, Electronic Supplementary Material 1: Radiomic Features Generation.
