# Supplementary material for: The Primacy of High B-Value 3T-DWI Radiomics in the Prediction of Clinically Significant Prostate Cancer
Source: Diagnostics (Basel). 2021 Apr 21;11(5):739. doi: 10.3390/diagnostics11050739 (PMC8143289; doi:10.3390/diagnostics11050739)
Supplement: Supplementary file 1 [file diagnostics-11-00739-s001.zip › diagnostics-1153936-supplementary.pdf]

Electronic Supplementary Material 1

Radiomic Features Generation

First, the following 7 statistical descriptors were computed on *local* windows (LW) centred in each pixel x_i of the DWI_{b2000} Region of Interest (ROI), whether ROI is the PCa lesion, thus achieving 7 parametric maps (PM) of local features. In that case, the symbol of feature is followed by subscript 'L', standing for local. Second, 12 statistical descriptors, including the 7 abovementioned, were computed globally, on each of the whole 7 PM. $\boldsymbol{\Omega}$ represents the domain where the features were computed on, and it can be either a LW, or the whole PM, and N is the number of pixels in $\boldsymbol{\Omega}$. The in-house software used was written in MATLAB® (R2018b v.9.5, The MathWorks, Natick, MA, USA).

From 1. to 7., $\boldsymbol{\Omega}$: LW or whole PM, $x_i \in \Omega$, i=1..N

$$\boldsymbol{E_L, E}: \boldsymbol{entropy} = -\sum_i p_{x_i} log\, p_{x_i} \tag{1}$$

where p_{x_i} is the probability of each pixel value $x_i \in \boldsymbol{\Omega}$

$$\boldsymbol{S_L, S}: \boldsymbol{skewness} = \frac{\frac{1}{N}\sum_i (x_i - \bar{x})^3}{\left(\frac{1}{N}\sum_i (x_i - \bar{x})^2\right)^{3/2}} \tag{2}$$

$$\boldsymbol{K_L, K}: \boldsymbol{kurtosis} = \frac{\frac{1}{N}\sum_i (x_i - \bar{x})^4}{\left(\frac{1}{N}\sum_i (x_i - \bar{x})^2\right)^{2}} - 3 \tag{3}$$

$$\boldsymbol{m_L, m}: \boldsymbol{mean} = \frac{1}{N}\sum_i x_i \tag{4}$$

$$\boldsymbol{M_L, M}: \boldsymbol{median} = x_{\left\lceil \frac{fc}{2} \right\rceil},\ f_c = \frac{\sum_i N p_{x_i}}{2} \tag{5}$$

$$\boldsymbol{CV_L, CV}: \boldsymbol{Coefficient\ of\ Variation} = \frac{\frac{1}{N}\sum_i x_i}{\sqrt{\frac{\sum_i (x_i - \bar{x})^2}{N}}} \tag{6}$$

$$\boldsymbol{IQR_L, IQR}: \boldsymbol{InterQuartile\ Range} = median_{upper\ half} - median_{lower\ half} \tag{6}$$

The following 5 statistical descriptors were computed globally on each of the 7 PM. From 8. to 12., $\boldsymbol{\Omega}$: PM.

(1) **MAX**: **Maximum value** = $\max_{x_i}(\Omega)$

(2) $\boldsymbol{\sigma}$: $\boldsymbol{standard\ deviation} = \sqrt{\frac{\sum_i (x_i - \bar{x})^2}{N}}$

(3) $\boldsymbol{MAD}$: $\boldsymbol{Median\ Absolute\ Deviation} = median(|x_i - median(\Omega)|)$

(4) $\boldsymbol{m_{90th}}$: $\boldsymbol{mean\ over\ the\ 90th\ percentile} = mean(x \in x_i \geq x_{90th})$

(5) $\boldsymbol{M_{90th}}$: $\boldsymbol{median\ over\ the\ 90th\ percentile} = median(x \in x_i \geq x_{90th})$
